# Assessment of Differential Item Functioning in Health-Related Outcomes: A Simulation and Empirical Analysis with Hierarchical Polytomous Data

**DOI:** 10.1155/2017/7571901

**Published:** 2017-09-12

**Authors:** Zahra Sharafi, Amin Mousavi, Seyyed Mohammad Taghi Ayatollahi, Peyman Jafari

**Affiliations:** ^1^Department of Biostatistics, Faculty of Medicine, Shiraz University of Medical Sciences, Shiraz, Iran; ^2^Department of Educational Psychology and Special Education, College of Education, University of Saskatchewan, Saskatoon, SK, Canada

## Abstract

**Background:**

The purpose of this study was to evaluate the effectiveness of two methods of detecting differential item functioning (DIF) in the presence of multilevel data and polytomously scored items. The assessment of DIF with multilevel data (e.g., patients nested within hospitals, hospitals nested within districts) from large-scale assessment programs has received considerable attention but very few studies evaluated the effect of hierarchical structure of data on DIF detection for polytomously scored items.

**Methods:**

The ordinal logistic regression (OLR) and hierarchical ordinal logistic regression (HOLR) were utilized to assess DIF in simulated and real multilevel polytomous data. Six factors (DIF magnitude, grouping variable, intraclass correlation coefficient, number of clusters, number of participants per cluster, and item discrimination parameter) with a fully crossed design were considered in the simulation study. Furthermore, data of Pediatric Quality of Life Inventory™ (PedsQL™) 4.0 collected from 576 healthy school children were analyzed.

**Results:**

Overall, results indicate that both methods performed equivalently in terms of controlling Type I error and detection power rates.

**Conclusions:**

The current study showed negligible difference between OLR and HOLR in detecting DIF with polytomously scored items in a hierarchical structure. Implications and considerations while analyzing real data were also discussed.

## 1. Introduction

In psychological, educational, and medical quality of life studies, measurement equivalence is an essential assumption for meaningful comparison of scores across different groups. Measurement nonequivalence can occur at different levels such as instrument or item level resulting in noncomparable data across groups [1–4]. The latter, which is known as differential item functioning (DIF), is an important part of a validation study. DIF analysis which originated in educational testing has been used in psychometric studies to assess whether the probability of responding to a specific item exhibits different statistical properties for different identifiable groups after controlling the construct being measured [1, 4]. There are two forms of DIF known as uniform and nonuniform. Uniform DIF is defined as a constancy of differences in the probability of correct answer for manifest group at all ability levels. In nonuniform DIF or crossing DIF (CDIF), the direction of such difference changes at some ability levels leading to different directions of DIF along the ability scale [5–10]. These forms become more complex when considering polytomous items rather than dichotomous [11]. Methodology reviews showed that there are several parametric and nonparametric statistical methods for evaluating DIF with dichotomous and polytomous items [9, 11, 12]. Among all these available methods, item response theory (IRT) [13] and ordinal logistic regression (OLR) [7] approaches have received notable attention in research and applied settings for polytomous items [2, 12]. IRT is the most powerful DIF detection approach, but it requires large sample size especially in case of models with more than one parameter such as two- and three-parameter models. Previous studies showed that for sufficient power when assessing DIF across two group a total sample size of 1000 for two groups is necessary [14]. Furthermore, the common unidimensional IRT models have strict assumptions (e.g., unidimensionality and local independency) compared to OLR. The OLR DIF detection approach is an effective, easy to implement and model-based procedure that it does not assume normality for the ability and can control additional covariates, both categorical and continuous, which may confound the results of DIF analysis. Moreover, this method provides a number of criteria to quantify the magnitude of DIF [5, 6, 12, 15, 16]. Multilevel or hierarchical data structure often arises from most of the common sampling designs used in educational and medical research. For example, total sample size in almost any public health-related survey is a combination of patients that are nested within healthcare service providers nested within districts. The districts can also be further nested within higher level units such as provinces/states [9, 17]. People nested in the same cluster share the same cluster-specific influences. Ignoring such cluster-specific influences can lead to cluster level unobserved heterogeneity and dependence between responses for participants in the same cluster [18]. Multilevel models simultaneously handle respondent level relationship and take account of this unobserved heterogeneity and dependence [6, 17, 19]. In the case of DIF analysis, the analytic strategy should also match the data structure [6]. Previous research has demonstrated that ignoring this structure may lead to inaccurate estimation of parameters and their standard errors, biased statistical tests, incorrect DIF detection, and inflates Type I error rate [6, 20].

Recent advancement in computing power and the availability of new software to fit multilevel models caused much attention paid to developing complicated models especially in DIF analysis [9]. There are several multilevel DIF detection simulation studies in the literature that have focused on dichotomously scored items [9, 20]. For example, Kamata et al. (2005) used generalized Rasch model with group membership to investigate detection of DIF [21]. In a series of studies, French and Finch expanded logistic regression, multiple indicator multiple cause (MIMIC), and Mantel–Haenszel models for detecting DIF in multilevel data [6, 22, 23]. Recently, Jin et al. (2013) studied the effect of item's and total score's intraclass correlation coefficient (ICC) on DIF, and Wen (2014) investigated DIF detection in both levels simultaneously. The results and implications of these studies, however, were limited to the dichotomous multilevel data.

To date, based on the authors' knowledge, there has not been a simulation study that investigated the performance of hierarchical ordinal model in DIF detection, while in practice, a wide variety of psychological, educational, and medical (e.g., health-related quality of life questionnaire (HRQoL)) outcome variables are polytomously scored and measured using Likert types of rating scales in which respondents choose their level of agreement on a symmetric agree-disagree scale [2, 24].

As mentioned, OLR is one of the most popular polytomous models that is able to utilize all the information from each item response [7] but it can only accommodate respondent level (i.e., level 1) information data. As discussed above, if standard analyses are used while ignoring the multilevel structure, variation due to the levels of the data structure could be combined, leading to incorrect parameter estimates and inflation standard errors; it may also result in increased likelihood of finding DIF when DIF is not present [2]. Therefore, the main objective of this study was to investigate Type I error rate and detection power of hierarchical ordinal logistic regression model (HOLR) and OLR in detecting DIF by means of Monte Carlo simulation. Additionally, a real data example was also analyzed.

## 2. Methods

### 2.1. DIF Analysis Based on Hierarchical Ordinal Logistic Regression Model

The logistic regression analysis has been suggested for DIF assessment by several studies (e.g., Swaminathan and Rogers, 1990; French and Miller, 1996; and French and Finch, 2010). Detecting DIF through OLR is based on comparing three different nested models. The full model as given by French and Miller (1996) has the following form:

(1) ηi=ln⁡pYi≤k ∣ g,θ1−pYi≤k ∣ g,θ=β0+β1θ+β2g+β3g∗θ,

where *p*(*Y*_*i*_ ≤ *k*) is the probability of responding at or below category* k* to an item for the* i*th person,*θ* represents ability and it is measured by the total test score, *g* is a grouping variable, and *g∗θ* represents the interaction between grouping variable and ability. The baseline model (*R*_1_) is a model that only includes the ability term. The next nested model (*R*_2_) includes the ability term plus the grouping variable as predictors. The value of the difference in −2 log-likelihood of full model and *R*_1_ can be used to detect uniform and nonuniform DIF simultaneously. This value can be compared to a Chi-square distribution with two degrees of freedom. If this comparison yields a significant result, the item is flagged for DIF and then further investigations are needed to test whether there is uniform or nonuniform DIF. Comparison between *R*_1_ and *R*_2_ can be utilized to assess uniform DIF [2].

As pointed out by French and Finch (2010), the primary problem of employing standard statistical models such as ordinal logistic regression with nested data is the violation of the assumptions of independently and identically distributed scores [6]. Although point estimates of parameter will not be seriously affected by this violation, estimate of standard errors, however, can be affected by dependency of data [6, 20], because nestedness of data or cluster sampling influences the sampling variance directly [2]. Inaccurate estimation of standard errors occurs in this type of data when the multilevel structure is not taken into account so that capturing the actual sampling variance should consider cluster sampling into account.

The above-mentioned DIF detection approach can be easily extended in order to accommodate multilevel data. The general model for the logit of responding at or below category* k* to an item for the* i*th person (e.g., student) in the* j*th cluster (e.g., school) for two levels can be written as

(2) level  1:  ηij=ln⁡pYij≤k ∣ Xqij,wsj1−pYij≤k ∣ Xqij,wsj=β0j+β1jX1ij+β2jX2ij+⋯+βqjXqijlevel  2:  βqj=γq0+∑s=1sqγqswsj+uqj,

where *Y*_*ij*_ is the polytomous item response for person* i* in cluster* j*; *X*s are person level predictors; *w*s are cluster level predictor; and *β* and *γ* are the associated regression coefficients for *X* and *w*, respectively. *u*_*qj*_ is random effect at cluster level. This general model for uniform DIF for within-cluster variable can be reduced to the following:

(3) level  1:  ηij=ln⁡pYij≤k ∣ θij,Gij1−pYij≤k ∣ θij,Gij=β0j+β1jθij+β2jGijlevel  2:  β0j=γ00+u0j,β1j=γ10,β2j=γ20.

As mentioned before, *Y*_*ij*_ and *θ*_*ij*_ are the polytomous item response and ability for person* i* in cluster* j. G*_*ij*_ represents the group identifier for which DIF will be tested for person* i* in cluster* j*. When the regression coefficient *β*_2*j*_ in the above equation is significant in comparison with the baseline model, then the studied item will be flagged as showing uniform DIF. By the same logic, for between-cluster variable we have

(4) level  1:  ηij=ln⁡pYij≤k ∣ θij,Gj1−pYij≤k ∣ θij,Gj=β0j+β1jθijlevel  2:  β0j=γ00+γ01Gj+u0j,β1j=γ10,

where *G*_*j*_ represents the group identifier for which DIF will be tested in cluster* j*. If the regression coefficient *γ*_01_ in the above equation is significant compared to the baseline model, then the studied item shows uniform DIF at the cluster level.

### 2.2. A Simulation Study

A simulation study was conducted to evaluate Type I error rate and detection power of hierarchical ordinal logistic regression model and OLR in detecting items expressing uniform DIF. Six manipulated factors were included in the current Monte Carlo study to investigate the comparative performance of HOLR and OLR in identifying the rate of correct (i.e., detection power) and incorrect (i.e., Type I error) uniform DIF items.

### 2.3. Design of the Simulation Study

Following Wood (2011), a test containing 16 simulated items (15 core items and 1 studied item) for two groups (focal and reference) was generated using the R package for statistical computing [25]. Each item had five possible response categories. Item scores were generated using the multilevel graded response model as given by Fox (2007) [19]. In each test, item difficulty parameters were sampled from the uniform distribution over interval [−2.5, 2.5]. In each simulation, values of ability for two groups were derived simultaneously from a 2-level normal linear mixed model as given by Wen (2014) and Jin et al. (2013) [9, 20]. Other factors considered in this study are as follows.

#### 2.3.1. Grouping Variable

As in French and Finch (2010, 2011, 2013) and Jin et al. (2013), two types of grouping variable were dichotomously simulated which are within cluster (e.g., gender or age group) and between cluster (e.g., type of school or teaching method). DIF at the between cluster was generated based on the idea that school effectiveness might impact performance on particular items which may increase or decrease the number of DIF items [9]. For simplicity, only one dichotomous grouping variable was included at each level and DIF was presented at the person level, although more than two groups and more characteristics and levels can be included to identify potential sources of DIF [9, 20]. Moreover, from previous DIF simulation work, equal or unequal comparison group sample sizes threaten Type I error rate and power in LR DIF analyses [26] Thus, in this study, at both levels a balanced design was considered.

#### 2.3.2. Intraclass Correlation Coefficient (ICC)

Three magnitudes of ICC (0.05, 0.25, and 0.45) were selected that present small, medium, and large correlations between individuals within each cluster. This selected range is in accord with range of ICC that is seen in applied educational and recent simulation studies [6, 20, 22, 23].

#### 2.3.3. Number of Clusters

Number of clusters is a critical factor that can affect both Type I error rate and power. Previous studies have determined that a greater number of clusters result in larger power and smaller Type I error rates [9]. Fifty groups are a frequently occurring number of clusters in practice in school research [27]. Therefore, in this study, the numbers of clusters simulated were 50, 100, and 200.

#### 2.3.4. Number of Participants per Cluster

The numbers of participants per cluster were 5, 10, and 20. These values are in accordance with previous research on hierarchical data.

#### 2.3.5. DIF Magnitude

Mild and severe uniform DIF were also simulated by adding 0.4 and 0.8 to the difficulty parameters of the focal group examinees, while the reference group examinees item difficulty parameter remained unchanged [9]. These levels were based on prior DIF simulation work with LR and hierarchical logistic regression [6, 12].

#### 2.3.6. Item Discrimination Parameters

Previous simulation studies have shown that high item discrimination parameter can result in highly inflated Type I error and high detection power [28]. Pervious research also showed that the inflation of Type I error rate was controlled fairly well when the discrimination parameters have a small range in LR DIF detection method [29]. Thus, for the purpose of this study, item discrimination parameters were sampled from the uniform distribution over two intervals as low (0.5–0.99) and high (1.5–2) discrimination parameters.

The analysis of OLR and HOLR also was conducted using R and Type I error rate and detection power were computed under the simulated conditions. Each condition was replicated 1000 times. In order to determine which manipulated factors influenced these rates, four repeated measure ANOVAs were conducted where Type I error and detection powers averaged across replications for a given condition were considered as dependent variables, the repeated measures factor was type of test (i.e., OLR or HOLR), and the manipulated factors were between-subjects factors. In addition to the tests of significance, the effect size (partial *η*^2^) was also calculated for each factor.

## 3. Results

### 3.1. Within-Cluster Variable

A repeated measures ANOVA was conducted to identify the factors that were significantly associated with the rate of false DIF identification (i.e., Type I error rate). This analysis showed that the highest significant effects with reasonable partial effect size were the interaction between the number of clusters and method (*p* value < 0.001 and *η*^2^ = 0.359) and interaction between the sample size per cluster and method (*p* value < 0.001 and *η*^2^ = 0.609). The standard way to interpret an interaction is to assess the effect of one factor at each level of other factors. Simple main effects of method were significant at sample sizes 5, 10 and cluster size 50. In all these three cases (sample sizes 5, 10 and cluster size 50), Type I error rate of HOLR was higher than OLR.

**Figure 1:**
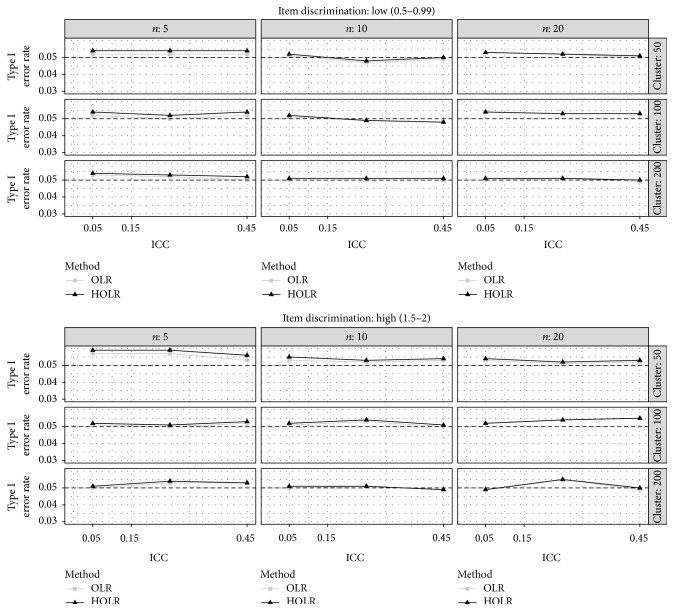
Type I error for within-cluster DIF variable by method, ICC, sample size per cluster, number of cluster, and item discrimination. The horizontal black dashed line represents the nominal alpha level of 0.05.

Figure 1 illustrates Type I error rates across manipulated factors for both OLR and HOLR when DIF occurs for within-cluster variable. Across almost all conditions, lines are nearly overlapped. The only exceptions were for the condition with 50 clusters and 5 cases per cluster and the condition with low item discrimination, 100 clusters, and 5 cases per cluster in which OLR outperformed HOLR.

This means in these conditions that the OLR showed closer estimates to the nominal Type I error rate of 0.05 compared to HOLR. Nevertheless, both OLR and HOLR controlled the nominal Type I error rate of 0.05 reasonably well and the maximum difference of two methods was as small as 0.002.

The detection powers for OLR and HOLR by manipulated factors, when DIF occurs for within-cluster variable, are shown in Table 1. As revealed, the detection power for both approaches in high item discrimination and high level of DIF was one and in most of the conditions, this was above the acceptable rate of 0.8 [20]. The exceptions were for the condition with low level of DIF (i.e., 0.4), 50 clusters, and 5 cases per cluster and the condition with low item discrimination, low level of DIF, and total sample size equal to or less than 500 in which the detection power rate was ranging from 0.303 to 0.784. In addition, the OLR method was less powerful than HOLR across almost all conditions, with maximum difference between the two methods being as small as 0.006. Table 1 clearly shows that detection power for both approaches increased with larger cluster sizes and more clusters. Detection power also increased with higher levels of DIF and discrimination parameter. Additionally, this increase in power across sample size per cluster and the number of clusters were more pronounced for low level of DIF and low discrimination parameter compared to higher levels of DIF and discrimination parameter. As with the detection power the repeated measures ANOVA revealed that the interaction between the method, item discrimination parameter, DIF magnitude, number of clusters, and cluster sample size was significant (*p* value < 0.001 and *η*^2^ = 0.382). The results reveal a significant 5-way interaction that is difficult to interpret.

### 3.2. Between-Cluster Variable

Similar to the within-cluster variable, repeated measures ANOVA was conducted in order to analyze Type I error rate for between-cluster condition. The interaction between number of clusters and method was significant (*p* value < 0.001 with *η*^2^ = 0.763). Simple main effect of method was significant with large partial effect size at each level of number of clusters. Furthermore, the main effect of sample size per cluster (*p* < 0.001, partial *η*^2^ = 0.355) was also statistically significant.

**Figure 2:**
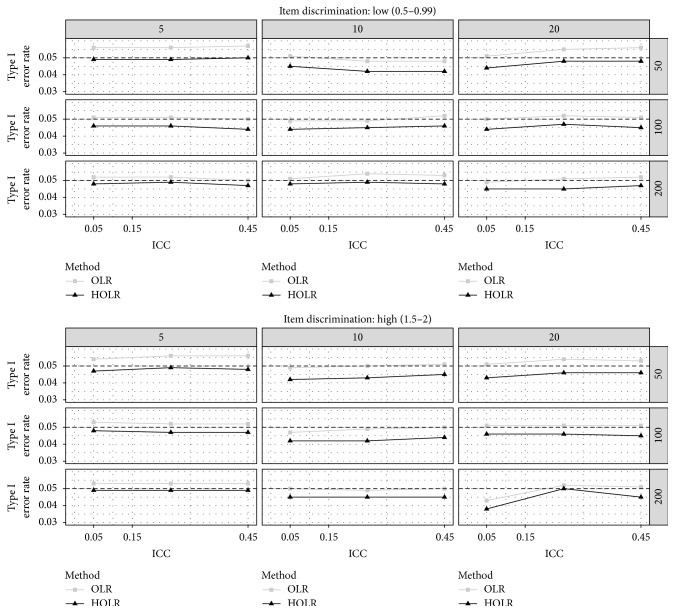
Type I error for between-cluster DIF variable by method, ICC, sample size per cluster, number of cluster, and item discrimination. The horizontal black dashed line represents the nominal alpha level of 0.05.

Figure 2 also illustrates Type I error rates across manipulated factors for both OLR and HOLR when DIF occurs for between-cluster variable. Across almost all conditions, the OLR showed closer estimates to the nominal Type I error rate of 0.05 compared to HOLR. The only exceptions were for the condition with 50 clusters and 5 cases per cluster and the condition with high item discrimination, 200 clusters, and 5 cases per cluster in which HOLR outperformed OLR. Another condition that HOLR performed slightly better than OLR was the condition with low item discrimination, 200 clusters, and 10 cases per cluster. It seems that the value of ICC affected Type I error rate only for larger sample sizes and mostly for HOLR. Similar to within-cluster variable, both OLR and HOLR controlled the nominal Type I error rate of 0.05 reasonably well and the magnitude of differences in Type I error rate across the two methods was again negligible (i.e., 0.003 to 0.006).

Table 2 shows the detection power of the two approaches for between-cluster variable. In contrast to the case for within-cluster variable, across almost all conditions the power for HOLR was lower compared to OLR. Similar to within-cluster variable, almost power of both approaches was above the acceptable level of 0.8. As the number of clusters and sample size within each cluster increased, power increased. The power of both methods was lower for lower level of DIF magnitude and discrimination parameter. As with the detection power, a repeated measures ANOVA was used for further investigation, and the highest order significant interaction was method by number of clusters by sample size per cluster by level of DIF by item discrimination parameter (*p* value < 0.001, partial *η*^2^ = 0.457).

## 4. Discussion

DIF and validity of test, especially at the item level, are important issues in the assessment of educational and psychological questionnaires. It is important to ensure that latent traits of all examinees were accurately determined by items and test scores. Although many studies have been done to expand DIF detection methods to polytomously scored items [23] and great attention also has been given to multilevel ordinal logistic regression, relatively few of them have focused on hierarchical polytomous DIF that is often present in a variety of psychological, medical, and educational researches.

As emphasized by French and Finch (2010, 2011, and 2013), ignoring the multilevel structure of data will result in the differences in DIF detection power rates when the analytic strategy does not match the data structure. The current study extended French and Finch's studies by considering polytomously scored items instead of dichotomous items. Based on our findings, HOLR and OLR performed almost equivalently in terms of controlling Type I error rate at the nominal alpha level of 0.05. The magnitude of Type I error rate's inflation of OLR as compared to HOLR, although negligible, for the between-cluster variable was not as small as the within-cluster variable.

This finding is in line with Jin et al. (2013) results. As compared to French and Finch (2010, 2011, and 2013), the results of the current study were similar to theirs for within-cluster variable, where methods performed similarly in terms of maintaining Type I error rate at the nominal level. However, the magnitude of Type I error rate inflation of OLR as compared to HOLR was not as large as what was found in other studies for the between-cluster variable. When cluster size and number of clusters were large enough, there were not any significant differences between Type I error rate of the two methods. This trivial difference across all levels of manipulated factors in the current study is consistent with prior research on the topic with dichotomous score, using different methods of DIF detection [6, 20, 22, 23]. The two methods maintained the power above the acceptable level (0.8) across most of the conditions. Power was extremely lower than 0.8 for sample sizes less than 500 with low level of DIF magnitude and item discrimination parameter across all levels of ICC.

For larger sample sizes, power was higher for both approaches. As with previous studies analyzing dichotomous items, power for both methods increased for large magnitudes of DIF and item discrimination parameter [6, 30, 31]. The results indicated that when high values of item discrimination and DIF magnitude are obtained, both methods can reliably identify items exhibiting DIF. Although the use of standard OLR DIF detection approach conceptually is inconsistent with the complex structure of the data, the results of this study demonstrated that using OLR may not lead to incorrect DIF detection, which is in line with Jin et al. (2013) results.

### 4.1. Application to Real Data

#### 4.1.1. Data

The Persian version of the Pediatric Quality of Life Inventor (PedsQL) 4.0 which measures health-related quality of life questionnaire in healthy children and adolescents was completed by 576 healthy school children (49.1% boys, 50.9% girls) and their parents in 16 schools (53.5% middle schools and 46.5% high schools). The PedsQL 4.0 Generic Core Scale, which had been translated and validated previously in Iranian population [16, 32, 33], consists of 23 items in four domains: physical health subscale (eight items), emotional functioning subscale (five items), social functioning subscale (five items), and school functioning subscale (five items). This measure uses a five-point Likert scale (“0 = never a problem, 1 = almost never a problem, 2 = sometimes a problem, 3 = often a problem, and 4 = almost always a problem”). The PedsQL 4.0 scoring protocol has used such that higher scores imply better HRQoL.

The participants were randomly selected by a two-stage cluster random sampling technique. In each of the four educational districts, four schools were selected at random (first stage). Once the schools were chosen, random number table was used to select two classes from each school randomly. All the students in the chosen classes were taken as studied sample (second stage).

Even though, in our simulation study, both OLR and HOLR approaches showed very similar results in DIF detection, in real world situations data might be influenced by other factors that were not considered in the simulation study. So, it is imperative to check the statistical assumptions before proceeding with data analysis. Intraclass correlation coefficient and design effect were calculated for each question in order to determine if a hierarchical analysis of the data is appropriate. The ICC and the design effect both indicate the need for multilevel modeling for this data. So, the hierarchical ordinal logistic regression DIF detection approach was used for detecting DIF in this data. Although ordinary logistic regression is frequently used to detect DIF in the field of health and quality of life research (e.g., [34]), to date, there has not been a study that utilizes multilevel modeling for detecting DIF in this area. Two different HOLR models were used for detecting DIF among gender (male = 0; female = 1), as the within-cluster variable and type of school (middle school = 0; high school = 1) as the between-cluster variable.

#### 4.1.2. Results

The results of the HOLR DIF analysis were summarized in Table 3. The estimated regression coefficients (*β*), result of Chi-square difference test, and corresponding *p* values are shown for all questions. While 11 out of 23 items (47.8%) showed uniform DIF among gender, just five items (21.7%) showed uniform DIF among two types of school.

**Figure 3:**
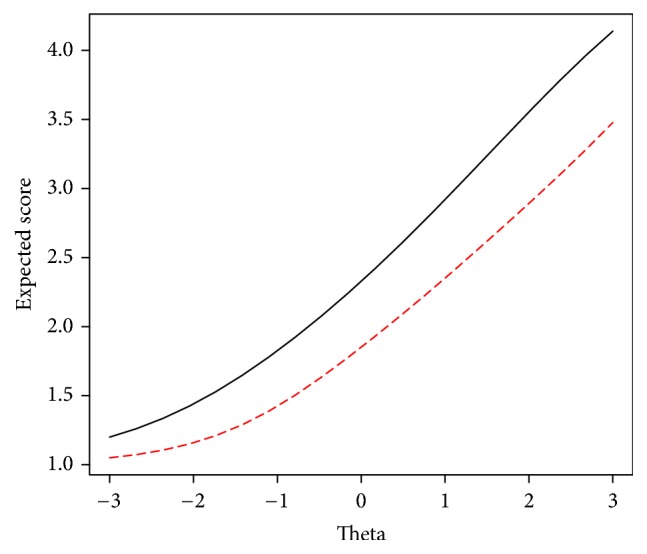
ICF of item (4) in physical health subscale, girl (solid line) and boy (dashed line).

Items (2), (3), and (4) in physical health, items (1) and (2) in emotional functioning, all items except the first item in social functioning, and items (1) and (3) in school functioning were flagged with DIF across gender. According to *β* values, items of social functioning and school functioning subscales were in favor of girls, whereas other items were in favor of boys. The item characteristic function (ICF) can be used as a summary statistic for a polytomous item especially in order to illustrate DIF. The ICF is defined as sum of the expected scores over response categories for each item (Nering and Ostini, 2011). When we have an item with *m*_*j*_ categories, ICF can be defined as the following formula:

(5) $$\begin{array}{c} E\left(\;X_{j}\;|\;\theta \;\right)=\overset{m_{j}}{\underset{x=0}{\sum }}xp_{jx}\left(\;\theta \;\right), \end{array}$$

where *p*_*jx*_(*θ*) is the probability of a score of *x* in the *j*th response category of item* X*.  Figure 3 depicts the ICF of item (4) in physical health in which girls (i.e., solid black line) have higher expected scores compared to boys (i.e., dashed line) even for lower theta (i.e., HRQoL) values.

**Figure 4:**
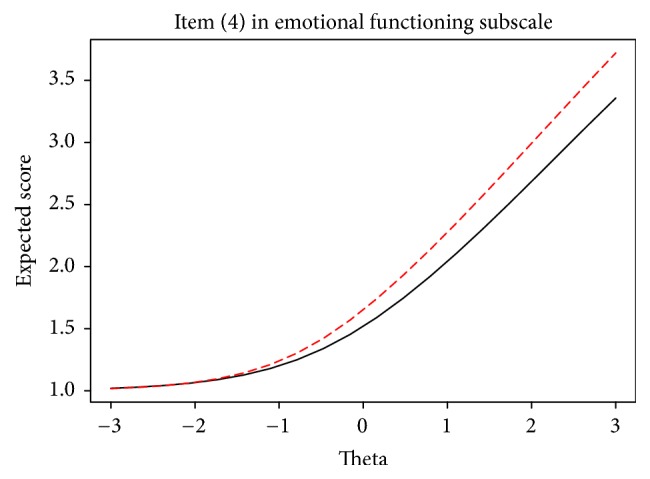
ICF of item (4) in emotional subscale, high school (solid line) and middle school (dashed line).

In terms of type of school, item (5) in physical health, item (4) in emotional and social functioning, and items (2) and (4) in school functioning subscales showed DIF across types of school. As it is shown in Table 3, item (4) in emotional functioning and item (2) in school functioning subscales were in favor of middle school students, whereas item (5) in physical health, item (5) in social functioning, and item (4) in school functioning subscales were in favor of high school students. It should be noted that item (5) in social functioning showed DIF across both within and between-cluster variables implying that female high school students are more likely to choose higher response category in this item.  Figure 4 shows the ICF of item (4) in school functioning in which high school students (i.e., dashed line) have higher expected scores compared to middle school students (i.e., solid black line) even for lower theta values.

## 5. Conclusion

As emphasized by French and Finch (2013) choosing proper modeling in analyzing hierarchical data is crucial because it allows for a potentially greater understanding of phenomena under study, as well as avoiding statistical misspecification. The current study extended previous studies by evaluating the comparative performance of HOLR and OLR in detecting differential item functioning for polytomous items. Results of the simulation study showed that when the grouping variable was at the within-cluster level, both OLR and HOLR performed equivalently in terms of controlling type I error rate at the nominal alpha level across almost all conditions. Interestingly, when the grouping variable was at between-cluster level, OLR showed closer Type I error rate estimates to the nominal alpha level of 0.05 compared to HOLR except for few conditions where HOLR outperformed OLR. With regard to detection power, detection power rates of both approaches were above the acceptable level of 0.8 with trivial differences across all simulation conditions. Despite the fact that this study found negligible difference between OLR and HOLR in detecting DIF with polytomously scored items, there are many unknowns while working with real data. Thus, as it was discussed earlier, it is necessary to check the tenability of statistical assumptions before choosing between OLR and HOLR.

As with other Monte Carlo simulation studies, results of this study are bounded by the factors under investigation, which limits generalization of the results. Additionally, choices made as to simulate a test with 16 items and only one DIF item, equal sample sizes for both reference, and focal groups at each level, considering DIF at each level separately and generating DIF only by adding a constant value to all threshold parameters of the studied item, may affect results of the simulation study. It has been shown that adding different values to threshold parameters can result in greater group differences across the continuum of ability (French and Miller, 1996). Additionally, in the simulation study, it was assumed that all category responses of items performed reasonably and effectively contributed to the results. This may not be true with real data and PedsQL has been found to have disordered categorical functioning [34]. It is also worth noting that we only used polytomous items but such simulation study can be easily extended to mixed-item format tests as a more realistic representation of reality.

We approached DIF solely from a hypothesis testing perspective in this study. As noted by other researchers, effect size measures are valuable tools in DIF. Further work examining DIF in polytomous items utilizing effect size measures is needed. Furthermore, we did not vary ability distribution throughout the simulation study, whereas it is very likely to observe different ability distributions between reference and focal groups as well as level 2 clusters. Assessing the effect of ability distribution on DIF is another area of investigation. After all, comparing relative performance of other methods of detecting DIF in polytomous data with hierarchical structure such as MIMIC modeling and PolySIB [35] would shed more light into this area of research.

## Figures and Tables

**Table 1:** Detection power for within-group variable by type of analysis and manipulated factors.

Variable			Item discrimination							
			Low (0.5–0.99)				High (1.5–2)			
*ρ*	Cluster	*N*	DIF = 0.4		DIF = 0.8		DIF = 0.4		DIF = 0.8	
			OLR	HOLR	OLR	HOLR	OLR	HOLR	OLR	HOLR
0.05	50	5	0.31	0.316	0.807	0.811	0.775	0.778	1	1
		10	0.559	0.564	0.986	0.987	0.976	0.976	1	1
		20	0.83	0.831	1	1	1	1	1	1
	100	5	0.519	0.523	0.977	0.977	0.966	0.966	1	1
		10	0.85	0.851	1	1	1	1	1	1
		20	0.984	0.984	1	1	1	1	1	1
	200	5	0.809	0.812	1	1	0.999	0.999	1	1
		10	0.988	0.989	1	1	1	1	1	1
		20	1	1	1	1	1	1	1	1

0.25	50	5	0.303	0.312	0.795	0.804	0.771	0.78	1	1
		10	0.556	0.558	0.988	0.988	0.977	0.977	1	1
		20	0.837	0.839	1	1	1	1	1	1
	100	5	0.533	0.54	0.976	0.977	0.971	0.972	1	1
		10	0.853	0.855	1	1	1	1	1	1
		20	0.981	0.981	1	1	1	1	1	1
	200	5	0.807	0.811	1	1	1	1	1	1
		10	0.99	0.99	1	1	1	1	1	1
		20	1	1	1	1	1	1	1	1

0.45	50	5	0.308	0.312	0.803	0.81	0.78	0.784	1	1
		10	0.558	0.561	0.989	0.99	0.975	0.977	1	1
		20	0.832	0.835	1	1	1	1	1	1
	100	5	0.533	0.537	0.98	0.98	0.971	0.972	1	1
		10	0.845	0.848	1	1	1	1	1	1
		20	0.984	0.984	1	1	1	1	1	1
	200	5	0.809	0.814	1	1	1	1	1	1
		10	0.991	0.991	1	1	1	1	1	1
		20	1	1	1	1	1	1	1	1

**Table 2:** Power for between-group variable by type of analysis and manipulated factors.

Variable			Item discrimination							
			Low (0.5–0.99)				High (1.5–2)			
*ρ*	Cluster	*N*	DIF = 0.4		DIF = 0.8		DIF = 0.4		DIF = 0.8	
			OLR	HOLR	OLR	HOLR	OLR	HOLR	OLR	HOLR
0.05	50	5	0.312	0.287	0.82	0.802	0.773	0.753	1	1
		10	0.555	0.529	0.982	0.981	0.971	0.966	1	1
		20	0.859	0.836	1	1	1	1	1	1
	100	5	0.571	0.55	0.979	0.977	0.975	0.973	1	1
		10	0.829	0.817	1	1	0.999	0.999	1	1
		20	0.982	0.979	1	1	1	1	1	1
	200	5	0.83	0.816	1	1	0.998	0.998	1	1
		10	0.984	0.981	1	1	1	1	1	1
		20	1	1	1	1	1	1	1	1

0.25	50	5	0.305	0.284	0.812	0.787	0.763	0.746	1	1
		10	0.55	0.51	0.982	0.972	0.967	0.955	1	1
		20	0.835	0.807	1	1	1	1	1	1
	100	5	0.557	0.53	0.979	0.975	0.975	0.968	1	1
		10	0.83	0.81	1	1	0.999	0.999	1	1
		20	0.971	0.963	1	1	1	1	1	1
	200	5	0.831	0.821	1	1	0.999	0.998	1	1
		10	0.985	0.983	1	1	1	1	1	1
		20	1	1	1	1	1	1	1	1

0.45	50	5	0.303	0.28	0.817	0.786	0.778	0.755	1	1
		10	0.534	0.487	0.983	0.976	0.971	0.964	1	1
		20	0.819	0.767	1	1	1	1	1	1
	100	5	0.551	0.525	0.98	0.977	0.974	0.969	1	1
		10	0.831	0.796	1	1	0.999	0.998	1	1
		20	0.974	0.954	1	1	1	1	1	1
	200	5	0.814	0.796	1	1	1	1	1	1
		10	0.979	0.969	1	1	1	1	1	1
		20	1	1	1	1	1	1	1	1

**Table 3:** Summary results from the HOLR method to identify differential item functioning on PedsQL TM 4.0 questionnaire.

	Gender			Type of school		
	*β*	Chi-square	*p* value	*β*	Chi-square	*p* value
Physical health						
(1)	0.463	3.111	0.08	0.247	1.07	0.3
(2)	0.476	8.02	**0.004**	0.025	0.021	0.9
(3)	0.656	10.234	**0.001**	−0.165	0.478	0.5
(4)	0.817	16.309	**<0.001**	0.327	1.707	0.2
(5)	0.811	2.422	0.1	1.115	7.488	**0.006**
(6)	0.237	2.179	0.1	−0.08	0.26	0.6
(7)	0.255	1.423	0.2	−0.33	2.49	0.1
(8)	0.164	0.767	0.4	−0.177	0.889	0.3

Emotional functioning						
(1)	0.468	7.508	**0.006**	0.188	1.075	0.3
(2)	0.688	10.183	**0.001**	−0.452	3.67	0.055
(3)	0.092	0.164	0.7	−0.298	2.598	0.1
(4)	−0.421	3.547	0.06	−0.587	8.273	**0.004**
(5)	0.029	0.002	0.97	−0.415	2.977	0.08

Social functioning						
(1)	−0.286	1.572	0.2	−0.208	0.819	0.4
(2)	−0.892	10.795	**0.001**	0.312	1.047	0.3
(3)	−1.307	21.365	**<0.001**	0.298	0.628	0.4
(4)	−0.449	5.836	**0.01**	0.07	0.144	0.7
(5)	−0.567	4.735	**0.02**	0.54	4.09	**0.04**

School functioning						
(1)	−0.518	4.748	**0.02**	−0.282	1.221	0.3
(2)	−0.137	0.691	0.4	−0.517	10.264	**0.001**
(3)	−0.939	11.538	**<0.001**	−0.482	2.35	0.1
(4)	−0.004	0.004	0.9	0.517	4.089	**0.04**
(5)	−0.194	0.755	0.4	0.334	2.14	0.14

Bold numbers represent the value of *p* value for items showing uniform DIF; Chi-square is the value of the difference in −2 log-likelihood of models 1 and 2, for testing uniform DIF.

## References

[1] Allahyari E., Jafari P., Bagheri Z. (2016). A Simulation Study to Assess the Effect of the Number of Response Categories on the Power of Ordinal Logistic Regression for Differential Item Functioning Analysis in Rating Scales. *Computational and Mathematical Methods in Medicine*.

[2] Jafari P., Sharafi Z., Bagheri Z., Shalileh S. (2014). Measurement equivalence of the KINDL questionnaire across child self-reports and parent proxy-reports: a comparison between item response theory and ordinal logistic regression. *Child Psychiatry and Human Development*.

[3] Bagheri Z., Jafari P., Tashakor E., Kouhpayeh A., Riazi H. (2014). Assessing whether measurement invariance of the KIDSCREEN-27 across child-parent dyad depends on the child gender: a multiple group confirmatory factor analysis. *Global journal of health science*.

[4] Mousavi A., Krishnan V. (2016). Measurement invariance of early development instrument (EDI) domain scores across gender and ESL status. *Alberta Journal of Educational Research*.

[5] Van De Water E. (2014). *A meta-analysis of Type I error rates for detecting differential item functioning with logistic regression and Mantel-Haenszel in Monte Carlo studies*.

[6] French B. F., Finch H. W. (2010). Hierarchical logistic regression: Accounting for multilevel data in DIF detection. *Journal of Educational Measurement*.

[7] French A. W., Miller T. R. (1996). Logistic regression and its use in detecting differential item functioning in polytomous items. *Journal of Educational Measurement*.

[8] Finch W. H., French B. F. (2007). Detection of crossing differential item functioning: a comparison of four methods. *Educational and Psychological Measurement. A Bimonthly Journal Devoted to the Development and Application of Measures of Individual Differences*.

[9] Wen Y. (2014). *DIF Analyses in Multilevel Data: Identification and Effects on Ability Estimates*.

[10] Bagheri Z., Jafari P., Faghih M., Allahyari E., Dehesh T. (2015). Testing measurement equivalence of the SF-36 questionnaire across patients on hemodialysis and healthy people. *International Urology and Nephrology*.

[11] Wood S. W. (2011). *Differential Item Functioning Procedures for Polytomous Items When Examinee Sample Sizes Are Small*.

[12] Narayanan P., Swaminathan H. (1996). Identification of items that show nonuniform DIF. *Applied Psychological Measurement*.

[13] Kim S.-H., Cohen A. S. (1998). Detection of differential item functioning under the graded response model with the likelihood ratio test. *Applied Psychological Measurement*.

[14] Tay L., Meade A. W., Cao M. (2015). An Overview and Practical Guide to IRT Measurement Equivalence Analysis. *Organizational Research Methods*.

[15] Crane P. K., Gibbons L. E., Jolley L., Van Belle G. (2006). Differential item functioning analysis with ordinal logistic regression techniques: DIFdetect and difwithpar. *Medical Care*.

[16] Jafari P., Ghanizadeh A., Akhondzadeh S., Mohammadi M. R. (2011). Health-related quality of life of Iranian children with attention deficit/hyperactivity disorder. *Quality of Life Research*.

[17] Peugh J. L. (2010). A practical guide to multilevel modeling. *Journal of School Psychology*.

[18] Skrondal A., Rabe-Hesketh S. (2004). *Generalized Latent Variable Modeling: Multilevel, Longitudinal, and Structural Equation Models*.

[19] Fox J.-P. (2007). Multilevel IRT modeling in practice with the package mlirt. *Journal of Statistical Software*.

[20] Jin Y., Myers N. D., Ahn S. (2013). Complex versus simple modeling for dif detection: when the intraclass correlation coefficient (*ρ*) of the studied item is less than the *ρ* of the total score. *Educational and Psychological Measurement*.

[21] Kamata A., Chaimongkol S., Genc E., Bilir K. Random-effect differential item functioning across group unites by the hierarchical generalized linear model.

[22] Finch W. H., French B. F. (2011). Estimation of {MIMIC} model parameters with multilevel data. *Structural Equation Modeling. A Multidisciplinary Journal*.

[23] French B. F., Finch W. H. (2013). Extensions of Mantel-Haenszel for Multilevel DIF Detection. *Educational and Psychological Measurement*.

[24] Jiang S., Wang C., Weiss D. J. (2016). Sample size requirements for estimation of item parameters in the multidimensional graded response model. *Frontiers in Psychology*.

[25] Team R.D.C R: A Language and Environment for Statistical Computing: the R Foundation for Statistical Computing.

[26] Herrera A.-N., Gómez J. (2008). Influence of equal or unequal comparison group sample sizes on the detection of differential item functioning using the Mantel-Haenszel and logistic regression techniques. *Quality and Quantity*.

[27] Maas C. J. M., Hox J. J. (2005). Sufficient sample sizes for multilevel modeling. *Methodology*.

[28] Hong T. The utility of the MIMIC model and MCFA method when detecting DIF using monte carlo simulation.

[29] Li Y. (2012). *Item discrimination and type I error rates in DIF detection using the mantel-haenszel and logistic regression procedures*.

[30] Chang H., Mazzeo J., Roussos L. (1995). Detecting dif for polytomously scored items: an adaptation of the sibtest procedure. *ETS Research Report Series*.

[31] Thurman C. (2009). *A monte carlo study investigating the influence of item discrimination, category intersection parameters, and differential item functioning patterns on the detection of differential item functioning in polytomous items*.

[32] Jafari P., Forouzandeh E., Bagheri Z., Karamizadeh Z., Shalileh K. (2011). Health related quality of life of Iranian children with type 1 diabetes: Reliability and validity of the Persian version of the PedsQL™ Generic Core Scales and Diabetes Module. *Health and Quality of Life Outcomes*.

[33] Jafari P., Bagheri Z., Ayatollahi S. M. T., Soltani Z. (2012). Using Rasch rating scale model to reassess the psychometric properties of the Persian version of the PedsQL TM 4.0 Generic Core Scales in school children. *Health and Quality of Life Outcomes*.

[34] Petersson C., Simeonsson R. J., Enskar K., Huus K. (2013). Comparing children’s self-report instruments for health-related quality of life using the International Classification of Functioning, Disability and Health for Children and Youth (ICF-CY). *Health and Quality of Life Outcomes*.

[35] Chang H.-H., Mazzeo J., Roussos L. (1996). Detecting DIF for polytomously scored items: An adaptation of the SIBTEST procedure. *Journal of Educational Measurement*.

[36] Sharafi Z. (2017). *An approach for testing measurement equivalence of psychometric measures with hierarchical polytomous data, in Biostatistics*.

